# Nomophobia (No Mobile Phone Phobia) and Psychological Health Issues among Young Adult Students

**DOI:** 10.3390/ejihpe13090128

**Published:** 2023-09-12

**Authors:** Nasrin Abdoli, Dena Sadeghi-Bahmani, Nader Salari, Mehdi Khodamoradi, Vahid Farnia, Somayeh Jahangiri, Annette Beatrix Brühl, Kenneth M. Dürsteler, Zeno Stanga, Serge Brand

**Affiliations:** 1 Substance Abuse Prevention Research Center, Health Institute, Kermanshah University of Medical Sciences, Kermanshah 6719851115, Iran; abdolinasrin511@yahoo.com (N.A.); mehdi0khodamoradi@gmail.com (M.K.); vahidfarnia@yahoo.com (V.F.); jahangirisomayeh84@gmail.com (S.J.); 2 Department of Psychology, Stanford University, Stanford, CA 94305, USA; bahmanid@stanford.edu; 3 Department of Epidemiology and Population Health, Stanford University, Stanford, CA 94305, USA; 4 Department of Biostatistics, School of Health, Kermanshah University of Medical Sciences, Kermanshah 6719851115, Iran; n_s_514@yahoo.com; 5 Sleep Disorders Research Center, Kermanshah University of Medical Sciences, Kermanshah 6719851115, Iran; 6 Center for Affective, Stress and Sleep Disturbances, Psychiatric Clinics of the University of Basel, 4002 Basel, Switzerland; annette.bruehl@upk.ch; 7 Division of Substance Use Disorders, Psychiatric Clinics of the University of Basel, 4002 Basel, Switzerland; kenneth.duersteler@upk.ch; 8 Department for Psychiatry, Psychotherapy and Psychosomatic, Psychiatric Hospital, University of Zurich, 8001 Zurich, Switzerland; 9 Centre of Competence for Military and Disaster Medicine, Swiss Armed Forces, 3008 Bern, Switzerland; zeno-giovanni.stanga@vtg.admin.ch; 10 Division of Sport Science and Psychosocial Health, Department of Sport, Exercise, and Health, Faculty of Medicine, University of Basel, 4052 Basel, Switzerland; 11 School of Medicine, Tehran University of Medical Sciences, Teheran 1417466191, Iran; 12 Center for Disaster Psychiatry and Disaster Psychology, Psychiatric Clinics of the University of Basel, 4002 Basel, Switzerland

**Keywords:** nomophobia, depression, anxiety, stress, obsessive–compulsive disorders, young adults

## Abstract

Background: Smart phone use has become a part of people’s everyday life. However, when the lack of using the smart phone to establish and maintain electronic communication is related to psychological distress, such a behavior may be considered a modern-age phobia, or nomophobia (no mobile phone phobia). The aims of the present study were to investigate among a sample of young adults the associations between scores for nomophobia and symptoms of depression, anxiety, stress, insomnia, and obsessive–compulsive disorders. Methods: A total of 537 students (mean age: 25.52 years; 42.3% females) participated in the study. They completed a booklet of self-rating questionnaires covering sociodemographic information and symptoms of nomophobia, depression, anxiety, stress, insomnia, and obsessive–compulsive disorders. Results: Higher scores for nomophobia were associated with higher scores for depression, anxiety, and stress, but not with scores for insomnia and obsessive–compulsive disorders. The regression model confirmed that symptoms of anxiety predicted nomophobia. Conclusions: The present results support the assumption that nomophobia appears to be a mood disturbance related to stronger associations with symptoms of anxiety and, to a lesser extent, with symptoms of depression and stress. By contrast, nomophobia appeared to be unrelated to insomnia and symptoms of obsessive–compulsive disorders.

## 1. Introduction

Smart phones belong to information and communication technologies (ICTs) and are an indispensable part of a person’s everyday life [1]. Compared to cell phones or mobile phones, which by definition allow prevalently sending texts and calling, smart phones allow for executing a broad variety of further tasks, such as texting and calling people; sending and reading emails; keeping private, leisure time, and working schedules synchronized; doing online shopping; managing social network sites (SNSs); gaming; executing workplace-related tasks [2]; and tracking one’s health behavior, including physical activity patterns, nutrition [3], sleep, and general health care, also termed Digital Health Technologies (DHTs; [4]). As such, it appears plausible that smart phones as a “working tool” have the power to enable learning, individual capacity, and human relationships [1], and to self-monitor health behavior. Besides the advantages of such smart phones for everyday life, questions arise as regards possible side effects, such as smart phone addiction [5,6,7,8,9,10,11,12], along with the question of whether and to what extent smart phones and their applications have the power to sustainably induce favorable health behavior, including sleep behavior [9,13,14,15,16], physical activity patterns [17,18,19], or smoking cessation [20,21,22], just to name but a few.

In this context, nomophobia (no mobile phone phobia) is considered a modern age-specific anxiety for not being able to communicate, for losing connectedness, for not being able to access information, and for giving up convenience [1]. The literature is not consistent as regards the wording and the underlying theoretical psychological concepts, in that some are talking about nomophobia [1,23,24,25,26,27,28], while some claim that excessive smart phone use is considered a smart phone addiction [5,9,10,12,29,30] or problematic or excessive smart phone use [6,11,31,32]. Further, to make the point in the case, nomophobia and excessive smart phone use were associated with higher scores for emotional loneliness and insomnia among a sample of 773 students (mean age: 25.95 years; 59.6% females) [29].

In the meanwhile, six meta-analyses and systematic reviews have addressed the associations between scores for nomophobia and possible mental health concerns.

Jahrami et al. (2022) [28] summarized data from 52 studies covering 47,399 participants from 20 different countries. The prevalence rates were 20% for mild nomophobia, 50% for moderate nomophobia, and 20% for severe nomophobia. Strikingly, students from non-Western countries were at increased risk of suffering from nomophobia, when compared to students from Western countries.

Osorio-Molino et al. (2021) [9] summarized data from 16 studies; the authors used the term nomophobia and smart phone addiction interchangeably and observed that higher scores for nomophobia/smart phone addiction were associated with higher scores for sleep disturbances and social distress and with lower scores for self-esteem and perceived social support.

Notara et al. (2021) [8] summarized data from 40 studies on young adults and reported prevalence rates for nomophobia ranging from 15.2% to 99.7%. Higher scores for nomophobia were associated with higher scores for social, psychological (depression, anxiety, stress), and social health issues, including somatic issues, such as higher scores for pain, fatigue, headache, and sleep.

León-Mejía et al. (2021) [24] summarized data from 102 studies and identified female gender and young age as risk factors for reporting higher scores for nomophobia. Further, the prevalence rates ranged from 13% to 79% of adults at risk, 6% to 73% of adults suffering from mild nomophobia, 25.7% to 73.3% of adults suffering from moderate nomophobia, and 1% to 87% of adults suffering from severe nomophobia.

Daraj et al. (2023) [27] summarized data from 16 studies among (young) adults. Higher scores for nomophobia were associated with higher scores for anxiety and smart phone addiction.

Last, Tuco et al. (2023) [23] summarized data from 28 studies covering 11,300 young adults from eight countries and reported the following prevalence rates: 24% for mild nomophobia, 56% for moderate nomophobia, and 17% for severe nomophobia.

To summarize the results of the meta-analyses and systematic reviews, the following findings are impressive: Since 2020, six meta-analyses and systematic reviews were published [8,9,23,24,27,28], reporting prevalence rates of nomophobia ranging from 1% to 87% of participants with severe nomophobia (e.g., [24]), summarizing data from 16 [9,27], 28 [23], 40 [8], 52 [28], and 108 studies [24], using nomophobia and smart phone addiction interchangeably [9], and associating higher scores for nomophobia with higher scores for symptoms of depression [8], anxiety [8,27], stress [8,9], and sleep disturbances [28] and lower scores for self-esteem and social support [9].

### The Present Study

We considered the overall results from the meta-analyses and systematic reviews and assessed dimensions of nomophobia, along with symptoms of depression, anxiety, stress, insomnia, and obsessive–compulsive disorders, though, and unlike reported in the above-mentioned meta-analyses and systematic reviews, we assessed all these dimensions in one single study. This approach is based on the transdiagnostic approach in psychiatry and clinical psychology [33,34,35,36,37], which indicated that the clinical presentation of individuals with symptoms of depression is such that they also report symptoms of anxiety and insomnia. In the same vein, and based on clinical and epidemiological surveys, approximately half of patients with the principal diagnosis of anxiety disorder also meet criteria for at least one additional comorbid psychiatric disorder. Given this, it appears plausible that symptoms of depression, anxiety, stress, and insomnia will be reported concomitantly. In addition to this, Oulasvirta, et al. [38] observed that excessive use of smart phones might be associated with compulsive behavior; given this, the decision was to additionally assess symptoms of obsessive–compulsive disorders.

Two hypotheses and two research questions were formulated. First, based on previous results, we expected that higher scores for nomophobia would be associated with higher scores for depression [8], anxiety [8,27], stress [8,9], and sleep disturbances [27]. Further, following the transdiagnostic approach [33,34,35,36,37] and the observation that symptoms of nomophobia might be associated with obsessive–compulsive behavior [38], we expected that symptoms of nomophobia would be associated with symptoms of obsessive–compulsive disorders. The first research question was: Which psychological dimensions (depression, anxiety, stress, insomnia, obsessive–compulsive disorders) could predict higher scores for nomophobia? The second research question was: Were prevalence rates of mild, moderate, and severe nomophobia similar or dissimilar to the prevalence rates of mild (20%), moderate (50%), and severe nomophobia (20%) reported in the systematic review and meta-analysis of Jahrami, et al. [28]?

## 2. Materials and Methods

### 2.1. Study Design

Students of the Kermanshah University of Medical Sciences (Kermanshah, Iran) were approached to participate in the present study. They were approached both via advertisements on the webpage of the faculty and via faculty members at the beginning of the first lectures. Students willing and able to participate scanned the specific QR code. On the first page of the online study, participants were fully informed about the aims of the study and the confidential and anonymous data handling. Thereafter, they signed the written informed consent. To do so, they ticked the specific box at the end of the first introductory page. Next, participants completed a series of self-rating questionnaires covering sociodemographic information and symptoms of nomophobia, depression, anxiety, stress, insomnia, and obsessive–compulsive disorders (see below). The study was performed between March and April 2023, thus, clearly, once COVID-19 pandemic-related social and educational restrictions were lifted. Further, the spring semester started shortly after Norooz (21 March). The Ethics Committee of the Vice-Chancellor for Research and Technology of the National Institute for Medical Research Development (NIMAD; Iran; ethic code: IR.NIMAD.REC.1400.159; registration number: 4000714) approved this study, which was conducted in accordance with the current revision [39] of the Declaration of Helsinki.

### 2.2. Participants

To test the hypotheses and to answer to the research questions, a total of 537 participants took part in the study; their mean age was 25.52 years (SD = 2.47); 227 (42.3%) were female; 474 (88.3%) were single, 354 (65.9%) were bachelor’s students, 118 (22.0%) were master’s students, and 65 (12.1%) were PhD students.

Table 1 reports all sociodemographic and psychological dimensions of the participants (the whole sample and separately for female and male participants.

Inclusion criteria were 1. Student of the Kermanshah University of Medical Sciences (KUMS; Kermanshah, Iran); 2. Age 18 years and older; 3. Willing and able to comply with the study conditions; 4. Signed written informed consent. Exclusion criteria were 1. Withdrew from the study; 2. Incomplete data; 3. Completing the questionnaires within about five minutes. Of the 600 students approached, 537 (89.5%) agreed to participating in the study and completed fully the questionnaires (see Table 1; Results section).

### 2.3. Measures

#### 2.3.1. Sociodemographic Information

Participants reported their age (years), sex at birth (female, male), marital status (single, married), highest degree (bachelor’s, master’s, PhD), and the duration of smart phone use per day (categories: 0–2 h; 3–4 h; 5–6 h; 7–more).

#### 2.3.2. Nomophobia Questionnaire (NMP-Q)

To assess nomophobia, participants completed the Farsi version [40,41] of the Nomophobia Questionnaire (NMP-Q) [1]. The questionnaire consists of 20 items. Typical items are “I would be worried because my family and/or friends could not reach me” [factor I: Not being able to communicate]; “I would be nervous because I would be disconnected from my online identity” [factor II: Losing connectedness]; “I would feel uncomfortable without constant access to information through my smartphone” [factor III: Not being able to access information]; and “Running out of battery in my smartphone would scare me“ [factor IV: Giving up convenience]. Answers are given on 7-point Likert scales ranging from 1 (= completely disagree) to 7 (= strongly agree), with higher sum scores reflecting a more pronounced nomophobia. The cut-off values are 0–20 = no nomophobia; 21–59 = mild nomophobia; 60–99 = moderate nomophobia; and 100–140 = severe nomophobia [1] (Cronbach’s alpha of the current study = 0.92; Cronbach’s alpha of the validated questionnaire = 0.945).

#### 2.3.3. Depression, Anxiety, Stress Scale-21 (DASS-21)

To assess symptoms of depression, anxiety, and stress, participants completed the Farsi version [42] of the Depression Anxiety Stress Scale [43]. It consists of 21 items. Typical items are “I felt down and depressed” [depression]; “I felt I was close to panic” [anxiety]; and “I was in a state of nervous tension” [stress]. Answers are given on 4-point Likert scales ranging from 0 (= does not apply to me at all) to 3 (= extremely applies to me), with higher sum scores reflecting a higher severity of symptoms. Accordingly, the subscale depression, the subscale anxiety, and the subscale stress were calculated separately (Cronbach’s alpha of the current study = 0.91 for depression, 0.92 for anxiety, and 0.90 for stress; Cronbach’s alphas of the validated questionnaire >0.71).

#### 2.3.4. Insomnia Severity Index (ISI)

To assess symptoms of insomnia, participants completed the Farsi version [44,45] of the Insomnia Severity Index (ISI) [46]. It consists of seven items asking, for instance, for the difficulty falling asleep and waking up early in the morning or being tired during the day. Answers are given on 5-point Likert scales ranging from 0 (= not at all) to 4 (= definitively), with higher sum scores reflecting a higher insomnia severity (Cronbach’s alpha of the current study = 0.90; Cronbach’s alpha of the validated study >0.81).

#### 2.3.5. Maudsley Obsessive–Compulsive Questionnaire (MOCI)

To assess symptoms of obsessive–compulsive disorders, participants completed the Farsi version [47] of the Maudsley Obsessive–Compulsive Inventory (MOCI) [48]. The questionnaire consists of 30 items. Typical items are “I spend a lot of time every day checking things over and over again” [factor Checking]; “I am often late because I can’t seem to get through everything in time” [factor Cleaning]; “I frequently get nasty thoughts and have difficulty in getting rid of them” [factor Slowness]; and “I usually have serious doubts about the simple everyday things I do” [factor Doubts]. Answers are given on dichotomous forced choices (yes = 1; no = 0), with higher sum scores reflecting a more pronounced severity of obsessive–compulsive behavior (Cronbach’s alpha of the current study = 0.86; Cronbach’s alpha of the validated study >0.71).

### 2.4. Statistical Analysis

A series of Pearson’s correlations was performed to investigate the associations between scores for nomophobia and age and scores for depression, anxiety, stress, insomnia, and obsessive–compulsive disorders.

To identify those factors predicting dimensions of nomophobia, a multiple regression analysis was performed with nomophobia as the dependent variable and symptoms of depression, anxiety, stress, insomnia, and obsessive–compulsive disorders as predictors. Preliminary conditions to perform a multiple regression analysis were met [49,50,51]: N = 537 > 100; predictors explained the dependent variables (R = 0.422, R^2^ = 0.18); the number of predictors: 5; 5 × 10 = 50 < N (537); and the Durbin–Watson coefficient was 1.53, indicating that the residuals of the predictors were independent. Further, the variances inflation factors (VIFs) were between 1.06 and 2.27; while there are no strict cut-off points to report the risk of multicollinearity, VIF < 1 and VIF > 10 indicate multicollinearity [50,51].

To compare prevalence rates of mild, moderate, and severe nomophobia reported in Jahrami et al. [28] with the current prevalence rates, an X^2^-test was performed.

The level of significance was set at alpha < 0.05. All statistical calculations were performed with SPSS^®^ 28.0 (IBM Corporation, Armonk, NY, USA) for Apple Mac^®^ (Cupertino CA, USA).

## 3. Results

### 3.1. Preliminary Calculations

With a series of single *t*-tests, we investigated whether female and male participants scored differently on age and symptoms of nomophobia, depression, anxiety, stress, insomnia, and obsessive–compulsive disorders. All ts were < 1.3, ps > 0.25; ds < 0.22. Accordingly, gender was not introduced as a confounder.

Further, age and duration of daily smart phone use were not associated with scores for nomophobia, depression, anxiety, stress, insomnia, and obsessive–compulsive disorders (all rs < 0.12, ps > 0.30). Given this, age and daily smart phone use were not further introduced as confounders.

Next, a first inspection of correlational computations revealed that there was no additional benefit running the statistics with the sub-dimensions of nomophobia (i.e., inability to communicate, communication loss, lack of access to information, loss of comfort and convenience) and obsessive–compulsive disorders (checking; washing; slowness; doubting), compared to the total scores for nomophobia and obsessive–compulsive disorders. Given this, the decision was to use the total scores for nomophobia and obsessive–compulsive disorders.

### 3.2. Associations between Nomophobia and Symptoms of Depression, Anxiety, Stress, Insomnia, and Obsessive–Compulsive Disorders

Table 2 reports the correlation coefficients (Pearson’s correlations) between dimensions of nomophobia and symptoms of depression, anxiety, stress, insomnia, and obsessive–compulsive disorders.

The results were as follows:

Higher scores for nomophobia were statistically significantly associated with higher scores for depression, anxiety, and stress. No associations were observed for insomnia and obsessive–compulsive disorders.

Higher scores for depression were statistically significantly associated with higher scores for anxiety, stress, and insomnia. No associations were observed for obsessive–compulsive disorders.

Higher scores for anxiety were statistically significantly associated with higher scores for stress. No associations were observed for insomnia and obsessive–compulsive disorders.

Higher scores for stress were statistically significantly associated with higher scores for insomnia. No associations were observed for obsessive–compulsive disorders.

Higher scores for insomnia and obsessive–compulsive disorders were not further associated.

Overall, the pattern of results was such that higher scores for nomophobia were associated with higher scores for depression, anxiety, and stress, but not with symptoms of insomnia and obsessive–compulsive disorders.

### 3.3. Predicting Scores for Nomophobia

To predict scores for nomophobia, a multiple regression analysis was performed. As mentioned, preliminary conditions to perform the multiple regression analysis were met (see Section 2.4. Statistical Analysis).

Table 3 reports the statistics of the multiple regression analysis. The only predictor for nomophobia was anxiety, while depression, stress, insomnia, and obsessive–compulsive disorders were excluded from the equation, as these variables did not reach statistical significance.

### 3.4. Categories of Nomophobia

To categorize the scores of the Nomophobia Questionnaire (NMP-Q), Yildirim and Correia [1] proposed the following categories: scores < 20: no nomophobia; 21 ≤ x ≤ 59: medium level of nomophobia; 60 ≤ x ≤ 99: moderate level of nomophobia; 100 ≤ x ≤ 140: severe nomophobia. Accordingly, 57 out of 537 (10.6%) had a medium level; 379 (70.6%) had a moderate level, and 101 (18.8%) had a severe level of nomophobia. Comparing prevalence rates as reported in Jahrami et al. [28] (mild nomophobia: 20%; moderate nomophobia: 50%; severe nomophobia: 20%), the test was: X^2^(N = 537, df = 4) = 6.58, *p* = 0.16.

## 4. Discussion

The aims of the present study were to investigate the associations between scores for nomophobia and symptoms of depression, anxiety, stress, insomnia, and obsessive–compulsive disorders among a sample of young adult students. The results showed that higher scores for nomophobia were related to higher scores for symptoms of depression, anxiety, and stress, but not to symptoms of insomnia or obsessive–compulsive disorders. Further, the regression model showed that anxiety was the only predictor for nomophobia. Last, the prevalence rates of mild, moderate, and severe nomophobia did not differ from prevalence rates reported in the recent meta-analysis [28]. The present results add to the current literature in the following four ways: First, we replicated and confirmed previous results reported in former meta-analyses; however, unlike previous studies, we assessed several symptoms concomitantly. Second, we showed that nomophobia was not related to symptoms of obsessive–compulsive disorders. Third, the only statistically significant predictor of nomophobia was anxiety. Fourth, the prevalence rates were statistically equal to former results, thus, further strengthening the reliability and validity of the present data.

Two hypotheses and two research questions were formulated, and each of these are considered now in turn.

With the first hypothesis, we expected that higher scores for nomophobia would be associated with higher scores for depression, anxiety, stress, and sleep disturbances, though data did not fully confirm these assumptions. To explain the present pattern of results and to associate this pattern with the current literature, the suggestions are as follows:

First, in accordance with previous studies, higher scores for nomophobia were associated with higher scores for depression [8], anxiety [8,27], and stress [8,9]. Thus, the present data confirm what is known in this specific field of research, though we expand upon previous research in that we assessed all dimensions of depression, anxiety, and stress concomitantly, while this was not the case for previous studies in the field. Two theoretical concepts may help to understand the associations between nomophobia, depression, anxiety, and stress:

First, the transdiagnostic approach in the field of clinical psychology and psychiatry [33,34,35,37,52] suggests that, for instance, symptoms of depression, anxiety, and stress are highly associated both because of their common neurophysiological basis of hyperarousal, including the up-regulated sympathetic system, and because of the high overlap of their symptoms. Second, the concept of allostatic load in well-being and ill-being suggests that a person per se might not suffer (well-being) or suffer (ill-being) from psychological distress [53,54]. In a more specific context, allostatic overload is understood as the cumulative effects of stressful experiences in daily life and may lead to disease over time [53,54,55]. Given this background, unsurprisingly, higher scores for nomophobia were associated with higher scores for depression, anxiety, and stress.

Second, as regards insomnia, no statistically significant association was found between nomophobia and insomnia. This observation was at odds with previous findings from naturalistic and observational studies [27], and also against the theoretical concepts of the transdiagnostic approach and the allostatic load.

To explain this gap, and thus greatly speculative, the following four assumptions are made.

First, to assess insomnia, we applied the Insomnia Severity Index (ISI; [45,46,56]), a highly standardized and validated self-rating questionnaire. As such, we assumed that previous studies in the field of nomophobia employed more coarse-grained, less specific measures, and with symptoms of sleep disturbances as the secondary research outcome [27]. However, a closer inspection of the meta-analysis and systematic review of Daraj et al. [27] showed that this assumption was incorrect in that the majority of studies reported in Daraj et al. [27] used the Insomnia Severity Index, too.

Second, given that nomophobia was related to symptoms of depression and anxiety, and given that symptoms of depression and anxiety are associated with higher scores for rumination and dysfunctional cognitive–emotional processes [57,58,59,60], questionnaires to assess pre-sleep procrastination [61,62,63,64,65,66,67] and sleep-related dysfunctional beliefs might have yielded more specific and indicative results. Future studies might consider this dimension of dysfunctional cognitive–emotional processes.

Third, as reported in Table 1, the insomnia mean score was 19.03, with a narrow standard deviation (2.79). This means that all participants were suffering from moderate clinical insomnia and that the variances were statistically too low to yield a more differentiated pattern of results. Given this, fourth, it appears that there was effectively no association between nomophobia and insomnia.

With the second hypothesis, we assumed that higher scores for nomophobia were associated with higher scores for obsessive–compulsive disorders, though data did not confirm this. Given this, the present results are at odds with previous findings of Oulasvirta et al. [38], who reported that symptoms of nomophobia might bear traits of compulsive behavior. We further claim that the present results do not match the theoretical concepts of the transdiagnosic approach [33,34,35,36,37,52] and of allostatic load [53,54,55]. The quality of the data does not allow a deeper understanding of the underlying psychological mechanisms to explain the zero-association between nomophobia and symptoms of obsessive–compulsive disorders. While again highly speculative, we advance the following assumption: From the viewpoint of evolutionary psychology and evolutionary psychiatry [68,69,70,71], symptoms of obsessive–compulsive disorders are the exacerbated and exaggerated endpoint of hygiene behavior to avoid physiological harm, in general, and psychological harm, more specifically. From this point of view, it appears plausible that dimensions of nomophobia and obsessive–compulsive disorders were both statistically and conceptually unrelated.

With the first research question, we asked which psychological dimensions (depression, anxiety, stress, insomnia, obsessive–compulsive disorders) could predict higher scores for nomophobia. The answer from the multiple regression analysis was that exclusively higher scores for anxiety predicted higher scores for nomophobia, while scores for depression, stress, insomnia, and obsessive–compulsive disorders did not reach statistical significance. Given this, it appears appropriate to consider nomophobia as an anxiety, in general, and as a specific fear, more specifically. However, unlike others (see [1]), for the following reasons, we do not claim that nomophobia should be introduced in international classification systems of psychiatric diseases, such as the ICD-11 [72] or the DSM-5-TR [73]. First, by definition, phobias developed over human evolution to cope with issues of survival and reproduction [68,69,70,71], while the observation of smart phone-related issues emerged within the last two decades with no association with survival and reproduction. Second, so far, nomophobia is exclusively assessed via self-reports, while a psychiatric diagnosis, by definition, needs a thorough clinical interview and robust experts’ ratings, followed by an experts’ consensus. Third, phobias have strong psychophysiological correlates, while, to our knowledge, no such research has been performed so far. Given this, future studies should assess psychophysiological markers to relate these to the experience of nomophobia. Further, a psychiatric classification requires a fully unambiguous terminology, which, however, is not the case so far: besides ‘nomophobia’ [1,23,24,25,26,27,28], others use ‘smart phone addiction’ [5,9,10,12,29,30] or ‘problematic or excessive smart phone use’ [6,11,31,32]. Fifth, a nosological category needs a clear description of the possible differential diagnostic overlap, which remains unresolved so far. Given these issues, we do not support the assumption that ‘nomophobia’ should be introduced as a nosological category.

The second research question was: Were prevalence rates of mild, moderate, and severe nomophobia similar or dissimilar to the prevalence rates of mild (20%), moderate (50%), and severe nomophobia (20%) reported in the systematic review and meta-analysis of Jahrami et al. [28], and the answer was yes. Given this, we claim that the present data appear highly reliable.

### Limitations

Despite the robust results, the following limitations should be considered. First, the sample consisted of young adult students, and such a sample does not reflect the general population. Second, and relatedly, all participants self-reported suffering at least from mild nomophobia, which, again, does not allow for transferring the current findings to the general population. Third, it is conceivable that further latent and, thus, unassessed dimensions, such as low self-esteem, high intolerance of uncertainty, and, above all, trait anxiety and social support, might have biased the current pattern of results. In this view, we may assume that symptoms of nomophobia might show a high overlap also with problematic internet use, and some more recent publications on this association appear to confirm this assumption [74,75,76,77,78]. In contrast, we may further assume that persons scoring high on resilience and mental toughness should be more protected from symptoms of problematic smart phone and internet use, given that both resilience [79,80,81,82,83] and mental toughness [84] are associated with higher scores for stress resistance. Fourth, the cross-sectional design of the study does not allow for understanding the direction of causality. Nevertheless, to run multiple regressions, an assumption of dependent and independent variables is necessary. To make a case in point, plausibly, we may assume that a basic trait of anxiety may lead to nomophobia and not vice versa. Clearly, longitudinal studies including further psychological dimensions mentioned above would help to understand the underlying psychological mechanisms, which may lead to nomophobic behavior. Fifth, the COVID-19 pandemic and its social restrictions showed us how important remote teaching, learning, and studying was. As such, it is also fully conceivable that a specific fear of missing online courses, meetings, workshops, and practice appears plausible. Given this, a more fine-grained assessment to understand why and for what purposes students used smart phones would have been helpful. Relatedly, sixth, future studies should assess the reasons for using the smart phone; plausibly, the psychological load differs when a person is concerned about pursuing university class and online exams, compared to following SNSs or to simply shop online.

## 5. Conclusions

The main aim of the present study was to test among adult university students, whether and to what extent symptoms of nomophobia were associated with symptoms of depression, anxiety, stress, insomnia, and obsessive–compulsive disorders. The key results were that symptoms of anxiety predicted scores for nomophobia, while, in contrast, symptoms of insomnia and obsessive–compulsive disorders appeared to be unrelated to nomophobia. Further, given the high prevalence rates of symptoms of nomophobia, and given that university students may use their smart phones for pursuing their studies, a more differentiated assessment appears mandatory.

## Figures and Tables

**Table 1:** Overview of the sociodemographic characteristics and the descriptive statistics of the symptoms of nomophobia, depression, anxiety, stress, insomnia, and obsessive–compulsive disorders for the whole sample and separately for male and female participants.

Variables		Total N (%)	Male N (%)	Female N (%)
Civil status	Single	474 (88.26)	284 (91.1)	190 (83.7)
	Married	63 (11.73)	26 (8.38)	37 (16.29)
	Total		310 (100)	227 (100)
Education	Bachelor’s	354 (5.9243)	210 (67.74)	144 (63.43)
	Master’s	118 (21.97)	68 (21.93)	50 (422.07)
	PhD	65 (12.10)	32 (10.32)	33 (14.53)
	Total		310 (100)	227 (100)
Average phone usage (time)	0–2 h	29 (5.4)	17 (5.48)	12 (5.28)
	3–4 h	67 (12.4)	35 (11.29)	32 (14.9)
	5–6 h	144 (26.8)	82 (26.45)	62 (27.31)
	7–8 h	297 (55.3)	176 (56.773)	121 (53.30)
	Total		310 (100)	227 (100)
	M (SD)		M (SD)	M (SD)
Age (years)	25.52 (2.46)		25.41 (2.39)	25.67 (2.56)
Depression Anxiety Stress Scale (DASS-21)	Depression	15.14 (3.94)	15.28 (4.002)	14.95 (3.84)
	Anxiety	14.76 (3.81)	14.58 (3.86)	14.64 (3.76)
	Stress	16.30 (3.69)	15.38 (3.69)	15.18 (3.71)
	Total score DASS	45.03 (9.87)	45.41 (9.91)	44.50 (9.82)
Insomnia Severity Index (ISI)		19.03 (2.79)	19.02 (2.75)	19.04 (2.84)
Nomophobia Questionnaire (NMP-Q)	Inability to communicate	25.33 (6.62)	25.29 (6.75)	25.37 (6.44)
	Losing connectedness	19.85 (60.90)	19.72 (6.43)	20.04 (5.59)
	Not being able to access information	18.93 (3.95)	18.96 (40.70)	18.90 (3.812)
	Giving up convenience	20.23 (5.924)	20.20 (6.090)	20.27 (5.702)
	Total score (NMP-Q)	84.35 (18.18)	84.17 (19.19)	84.59 (16.72)
Maudsley Obsessive–Compulsive Inventory (MOCI)	Washing	15.89(1.616)	15.90(1.657)	15.89(1.560)
	Slowness	10.23 (1.287)	10.23 (1.277)	10.23 (1.304)
	Doubting	9.59 (1.237)	9.76 (1.221)	9.36 (1.223)
	Checking	13.52 (1.405)	13.49 (1.398)	13.56 (1.417)
	Total score MOCI	43.33 (2.684)	43.48 (2.676)	43.11 (2.684)

**Table 2:** Correlation coefficients (Pearson’s correlations) between scores for nomophobia, depression, anxiety, stress, insomnia, and obsessive–compulsive disorders.

			Dimensions			
	Nomophobia	Depression	Anxiety	Stress	Insomnia	Obsessive–Compulsive Disorders
Nomophobia	-	0.34 ***	00.40 ***	0.32 ***	0.08	−0.03
Depression		-	0.63 ***	0.67 ***	0.21 **	0.02
Anxiety			-	0.60 ***	0.01	−0.00
Stress				-	0.13 **	0.09
Insomnia					-	0.05
Obsessive–compulsive disorders						-

Notes: ** = *p* < 0.01; *** = *p* < 0.001.

**Table 3:** Multiple linear regression with nomophobia as outcome variable and depression, anxiety, stress, insomnia, and OCD as predictors.

Dimension	Variables	Coefficient	Standard Error	Coefficient β	t	*p*	R	R^2^	Durbin–Watson
Nomophobia	Intercept	48.09	6.97	-	6.89	0.000	0.422	0.178	1.533
	Anxiety	1.405	0.265	0.287	5.312	0.000			
	Excluded variables: Insomnia, depression, stress, obsessive–compulsive disorders; t < 1.5; *p* > 0.16								

## Data Availability

Data are made available to explicit experts in the field. Such experts should clearly formulate their hypotheses; further, they should fully describe, how and where they do securely store the data file, and how they make sure that the data file is not shared with and securely protected from third parties.
